# Chicago Pathological Society

**Published:** 1883-04

**Authors:** I. H. Tebbetts

**Affiliations:** Secretary


### ARTICLE XIV.

### REPORT OF MEETING CHICAGO PATHOLOGICAL SOCIETY.

March 12, 1883. Society called to order by the president, Dr. Lyman.

The minutes of the last meeting were read and approved.

The following named gentlemen were elected to membership: Drs. J. J. M. Angear, C. A. Bucher, A. B. Bausman.

Dr. G. M. Hammond was proposed for membership by Drs. Bennett and Lyman, and Dr. J. M. Patton by Drs. Webster and Tebbetts.

Dr. A. Lagorio read a paper entitled "The Hypodermatic Treatment of Syphilis," which was discussed by the society.

Dr. G. Frank Lydston next read a paper entitled "The Treatment of Bubo," which was also discussed by the society.

The essaysists were requested, by the president, to offer their papers for publication in some journal.

Present, Drs. Newton, Taggert, Lyons, Hanson, Angear, Haight, Bennett, Lagorio, Lyman, Hammond, Lydston, Camp, Dobbin, Campbell, Tebbetts, and three visitors.

The society, on motion, adjourned.

I. H. TEBBETTS, *Secretary.*

---

PROF. DANFORTH'S clinic at St. Luke's Hospital, Thursdays, is at 1.30 P. M.

